# Thermoelectric Efficiency of a Topological Nano-Junction

**DOI:** 10.3390/e20050366

**Published:** 2018-05-14

**Authors:** Manuel Álamo, Enrique Muñoz

**Affiliations:** 1 Physics Institute, Pontificia Universidad Católica de Chile, Vicuña Mackenna 4860, Santiago 7820436, Chile; 2 Research Center for Nanotechnology and Advanced Materials CIEN-UC, Pontificia Universidad Católica de Chile, Vicuña Mackenna 4860, Santiago 7820436, Chile

**Keywords:** Keldysh formalism, topological superconductors, thermoelectricity, nano-junction

## Abstract

We studied the non-equilibrium current, transport coefficients and thermoelectric performance of a nano-junction, composed by a quantum dot connected to a normal superconductor and a topological superconductor leads, respectively. We considered a one-dimensional topological superconductor, which hosts two Majorana fermion states at its edges. Our results show that the electric and thermal currents across the junction are highly mediated by multiple Andreev reflections between the quantum dot and the leads, thus leading to a strong nonlinear dependence of the current on the applied bias voltage. Remarkably, we find that our system reaches a sharp maximum of its thermoelectric efficiency at a finite bias, when an external magnetic field is imposed upon the junction. We propose that this feature can be used for accurate temperature sensing at the nanoscale.

## 1. Introduction

Topology provides a fertile conceptual framework to many fields of modern physics, ranging from cosmology to condensed matter, but it is in the later where it has found its most powerful applications. Up to date, several topological phenomena have been discovered and studied in the context of solid state physics: The quantum spin Hall effect, topological insulators and Weyl semimetals, to name a few, and hence this constitutes a very active field of research.

An important class of topological materials which are currently gathering a lot of attention are topological superconductors (TS), mainly due to their potential application in topological quantum computers [1,2,3]. They were first proposed theoretically, in 2D [4] and 1D [5] models, where a spinless p-wave superconductor supports non-Abelian anyons or Majorana zero modes (MZM) in the vortex core in the 2D case [6], or at the edges in the one dimensional scenario. In principle, the presence of a Majorana bound state at the surface of a TS can be detected experimentally by the zero-bias anomaly [6,7,8,9,10]. In 2010 TS were experimentally fabricated [11,12] using a one dimensional semiconducting nano-wire with strong spin-orbit coupling, in proximity to an s-wave superconductor. Experimental evidence for the emergence of Majorana fermions in these systems has been reported [13]. Due to their nonlocal topological properties, Majorana fermions are protected against perturbations and different sources of local noise, thus representing a physical principle to implement fault-tolerant quantum computation.

While the electrical properties of topological materials have been intensively studied [6,7,8,9,11,12,14,15], thermoelectric performance of these topological based devices has not yet received the same level of attention from the condensed matter community, besides some interesting examples [15,16,17,18]. The heat production due to Joule heating in nano and micro-devices is one of the biggest problems in current microelectronics, and there is a growing interest on new materials capable of mitigating energy dissipation. In this context, proper thermal energy management and active cooling are nowadays highly required for the advance of new technologies [19,20,21,22]. In this work, we study a nano-device based on a topological superconductor, which can be used as a nano-thermocouple controlled by a weak magnetic field at small applied voltages. Our theoretical analysis suggests that this configuration can provide the basis for a highly sensitive temperature sensor, which can be used to detect Joule heating in micro-devices with nanometer scale resolution.

Along this article, we shall describe the transport properties and thermoelectric performance of a hybrid nano-junction formed by a quantum dot connected to an s-wave superconductor and a topological superconductor leads, respectively, as described in [14]. The Hamiltonian describing this model is presented in Section 2. The s-wave superconducting lead is submitted to a finite bias voltage, such that the BCS superconducting order parameter acquires a time-dependent Josephson phase, and therefore the junction is out of equilibrium. In Section 3 we apply the Keldysh formalism (described in Appendix A) to study the electrical current through our system. The current-voltage characteristics and the thermoelectric performance are presented in Section 4, for different externally applied magnetic fields. Finally, we conclude with the main discussion in Section 5. In Appendix B we provide the derivation of quantum-mechanical transport coefficients in the context of irreversible thermodynamics [23,24,25] and in analogy with Onsager reciprocal relations [24,26,27,28].

## 2. The Hybrid Nano-Junction

### The Model

**Figure 1:**
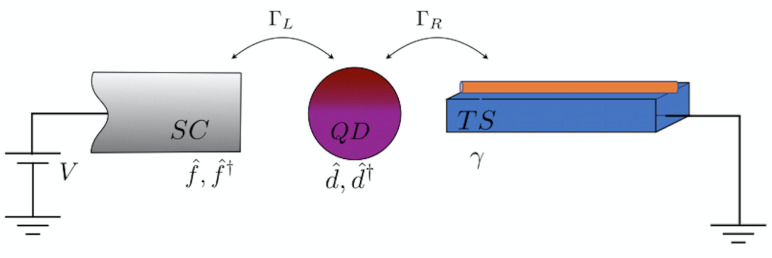
Sketch of the nano-junction.

The system that we shall study along this work was first proposed in [14]. As sketched in Figure 1, it consists on a hybrid nano-junction composed by a semiconductor quantum dot (QD), connected to a normal s-wave superconductor (SC) and a one dimensional topological superconductor (TS), that play the role of the left (L) and right (R) leads, respectively. The system is represented, in second quantization, by the Hamiltonian

(1) $$\hat{H}={\hat{H}}_{SC}+{\hat{H}}_{QD}+{\hat{H}}_{T}.$$

Due to the presence of the normal superconductor, the Hamiltonian is conveniently represented in spin and Nambu subspaces by a 4×4 matrix. The first contribution corresponds to the BCS Hamiltonian in the standard and symmetric Bogoliubov-de Gennes form

(2) $${\hat{H}}_{SC}=\frac{1}{2}\sum_{k}{\hat{f}}_{k}^{†}\left[\left(\begin{array}{cc} \xi_{k} & \Delta \\ \Delta^{*} & -\xi_{k} \end{array}\right)⊗{\hat{s}}_{0}\right]{\hat{f}}_{k},$$

where Δ is the BCS order parameter of the superconductor. On the other hand, the QD contribution is given by

(3) $${\hat{H}}_{QD}=\frac{1}{2}{\hat{d}}^{†}\left(\epsilon {\hat{\tau }}_{z}⊗{\hat{s}}_{0}+H{\hat{\tau }}_{0}⊗{\hat{s}}_{z}\right)\hat{d},$$

where $H=g\mu_{B}h$ is the Zeeman-coupling to an external magnetic field *h*, whereas ϵ is the local energy level at the quantum dot. Finally, the tunneling Hamiltonian, which couples the QD to the normal (*L*) and topological superconductor (*R*) leads via the coupling constants $\Gamma_{L},\Gamma_{R}$, respectively, is given by

(4) $${\hat{H}}_{T}=\frac{1}{2}\left(\Gamma_{L}{\hat{f}}^{†}\left(0\right){\hat{\tau }}_{z}⊗{\hat{s}}_{0}+\Gamma_{R}\gamma V^{†}{\hat{\tau }}_{z}⊗{\hat{s}}_{0}\right)\hat{d}+h.c.$$

Here, we have defined ${\hat{s}}_{\mu }$ and ${\hat{\tau }}_{\mu }$ ,with μ=0,1,2,3, the unit and Pauli matrices

(5) $$\begin{array}{c} {\hat{s}}_{0}={\hat{\tau }}_{0}=\left(\begin{array}{cc} 1 & 0\\ 0 & 1 \end{array}\right),\;\;\;\;{\hat{s}}_{1}={\hat{\tau }}_{1}=\left(\begin{array}{cc} 0 & 1\\ 1 & 0 \end{array}\right),\;\;\;\;{\hat{s}}_{2}={\hat{\tau }}_{2}=\left(\begin{array}{cc} 0 & -i\\ i & 0 \end{array}\right),\;\;\;\;{\hat{s}}_{3}={\hat{\tau }}_{3}=\left(\begin{array}{cc} 1 & 0\\ 0 & -1 \end{array}\right), \end{array}$$

acting on the spin ($\left\{{\hat{s}}_{\mu }\right\}$) and Nambu ($\left\{{\hat{\tau }}_{\mu }\right\}$) subspaces, respectively. In this spin-Nambu basis, the fermion operators ${\hat{f}}_{↑}$ and ${\hat{f}}_{↓}$ are organized in a 4-component vector $\hat{f}={\left(\begin{array}{cccc} {\hat{f}}_{↑} & {\hat{f}}_{↓} & {\hat{f}}_{↓}^{†} & -{\hat{f}}_{↑}^{†} \end{array}\right)}^{T}$, with an identical structure for the QD fermion operators ${\hat{d}}_{↑}$ and ${\hat{d}}_{↓}$. On the other hand, the Majorana operator γ is associated with the spinor $V_{\theta }={\left(\begin{array}{cccc} g_{\theta } & g_{\theta } & g_{\theta }^{*} & -g_{\theta }^{*} \end{array}\right)}^{T}$, where $g=e^{i\theta }$ is a constant phase [14]. For simplicity we will consider θ=0, and neglect the Coulomb interaction between electrons in the QD. Even though a TS hosts two Majorana Bound states (MBS) at each one of its ends, assuming a very long, semi-infinite lead, the tunneling problem can be reduced to that of transport through the SC-QD-MBS junction only.

## 3. Non-Equilibrium Transport Through the Nano-Junction

Let us consider a finite bias voltage *V* imposed upon the s-wave superconductor lead. In consequence, the BCS order parameter acquires a time-dependence $\Delta \to \left|\Delta \right|e^{i\phi (t)}$, where

(6) $$\phi \left(t\right)=\phi_{0}+2\frac{e}{\hbar }\int_{0}^{t}\;V\left(\bar{t}\right)d\bar{t}=\phi_{0}+2\frac{e}{\hbar }Vt$$

is a Josephson phase [7,14]. Because of this periodic time dependency, transport across the junction is highly non-linear and thus cannot be described by the standard Green-Kubo relations [23,29,30], which are strictly valid only for the linear response regime and in agreement with the fluctuation-dissipation theorem [29,30]. Therefore, transport in this quantum mechanical system must be analyzed with a formalism appropriate for non-equilibrium regimes, such as the reduced density matrix [7], equations of motion [15] or, as we chose in this work, non-equilibrium Green’s functions, also referred as the Keldysh formalism in condensed matter theory [24,25,31,32]. For readers which are not familiar with the method, we present a description of it in Appendix A.

For the nano junction described by the Hamiltonian Equation (1), the current operator is defined via continuity equation at the left SC lead by the expression

(7) $$\begin{array}{ccc} \hat{J} & = & e\frac{d}{dt}{\hat{n}}_{L}=\frac{e}{i\hbar }\left[{\hat{n}}_{L},\hat{H}\right]=-\frac{ie}{4\hbar }\Gamma_{L}\left({\hat{f}}^{†}\left(0\right){\hat{\tau }}_{3}⊗{\hat{s}}_{0}\hat{d}-h.c\right)\\ & \equiv & -\frac{ie}{4\hbar }\hat{j}, \end{array}$$

where ${\hat{n}}_{L}=\frac{1}{2}{\hat{f}}^{†}\left(0\right){\hat{\tau }}_{3}⊗{\hat{s}}_{0}\hat{f}\left(0\right)$ is the number operator of the conventional superconductor SC.

Starting from the full Hamiltonian Equation (1), we construct the effective action for the system on the Keldysh contour, following the argument presented in Equation (A16), by defining a source term involving the current in Equation (7)

(8) $${\hat{J}}_{s}=\frac{1}{4}\alpha \left(t\right)\hat{j}.$$

This source couples to the effective action via the first Pauli matrix ${\hat{\sigma }}_{1}=\left(\begin{array}{cc} 0 & 1\\ 1 & 0 \end{array}\right)$, thus breaking the symmetric time evolution on the Keldysh contour.

After integrating out the fermionic fields of the superconductor ($\hat{f}$) and the quantum dot ($\hat{d}$), we obtain an effective action in terms of the Green’s function of the Majorana fermions at the TS. Therefore, the reduced generating functional, including the source, can be expressed as

(9) $$Z_{C}\left[\alpha \right]=\int D\left[\gamma \right]\exp\left\{\frac{i}{2}\int \;dt\gamma^{T}{\hat{G}}_{TS}^{-1}\left[E,\alpha \right]\gamma \right\},$$

with ${\hat{G}}_{TS}^{-1}\left[E,\alpha \right]$ the Green’s function of the TS after the integration of the SC and QD fields. When expressed in the form of a Dyson equation, we have

(10) $${\hat{G}}_{TS}^{-1}\left[E,\alpha \right]={\hat{G}}_{TS,0}^{-1}\left(E\right)-{\hat{\Sigma }}_{TS}\left(E,\alpha \right),$$

with the TS self-energy given by

(11) $${\hat{\Sigma }}_{TS}\left(E,\alpha \right)={\left|\Gamma_{R}\right|}^{2}\left({\hat{\sigma }}_{0}⊗\left(V^{†}\cdot \left({\hat{\tau }}_{3}⊗{\hat{s}}_{0}\right)\right)\right){\hat{G}}_{QD}\left(E,\alpha \right)\left({\hat{\sigma }}_{0}⊗\left(\left({\hat{\tau }}_{3}⊗{\hat{s}}_{0}\right)\cdot V\right)\right),$$

and the non-interacting TS Green’s function components defined on the Keldysh space (*A*: Advanced, *R*: Retarded, *K*: Kinetic) as

(12) $${\hat{G}}_{TS,0}^{\left(R,A\right)}\left(E\right)=\frac{1}{E\pm i\eta }\;\mathrm{and}\;{\hat{G}}_{TS,0}^{K}\left(E\right)=0.$$

On the other hand, the QD’s Green’s function is given by

(13) $${\hat{G}}_{QD}\left(E,\alpha \right)={\left[{\hat{G}}_{QD,0}^{-1}\left(E\right)-{\left|\Gamma_{L}\right|}^{2}\left({\hat{\alpha }}_{-}{\hat{G}}_{SC}\left(E\right){\hat{\alpha }}_{+}\right)\right]}^{-1}.$$

Here, ${\hat{\alpha }}_{\pm }=\frac{1}{2}\left[{\hat{M}}_{030}\pm \alpha {\hat{M}}_{100}\right]$, ${\hat{M}}_{ijk}\equiv {\hat{\sigma }}_{i}⊗{\hat{\tau }}_{j}⊗{\hat{s}}_{k}$ with i,j,k={0,1,2,3} and the non-interacting QD Green’s function components on the Keldysh space are given by

(14) $${\hat{G}}_{QD,0}^{\left(R,A\right)}\left(E\right)={\left[\left(E\pm i\eta \right){\hat{\tau }}_{0}⊗{\hat{s}}_{0}-\epsilon {\hat{\tau }}_{3}⊗{\hat{s}}_{0}-H{\hat{\tau }}_{3}⊗{\hat{s}}_{3}\right]}^{-1}\;\mathrm{and}\;{\hat{G}}_{QD,0}^{K}\left(E\right)=0.$$

The QD and TS kinetic Green’s functions are identically zero, because in the absence of self-energy terms for these non-interacting subsystems, they are proportional to the complex regulator iη and hence they vanish in the limit $\eta \to 0^{+}$, in agreement with the fact that non-interacting systems are dissipationless [29,32]. Finally, the SC Green’s function in equilibrium (V=0) is

(15) $${\hat{G}}_{SC}^{\left(R,A\right)}\left(E\right)=A^{\left(R,A\right)}\left(E\right){\hat{\tau }}_{0}⊗{\hat{s}}_{3}+iB^{R,A}\left(E\right){\hat{\tau }}_{3}⊗{\hat{s}}_{2},$$

with A(E) and B(E) functions obtained from the standard BCS theory by [32]

(16) $$A^{\left(R,A\right)}\left(E\right)=\left\{\begin{array}{cc} \frac{-iE}{\sqrt{{\left|\Delta \right|}^{2}-E^{2}}} & |E|<\Delta \\ \frac{\pm E}{\sqrt{E^{2}-{\left|\Delta \right|}^{2}}} & |E|>\Delta \; \end{array}\right.\;,\;B^{\left(R,A\right)}\left(E\right)=\left\{\begin{array}{cc} \frac{-i\Delta }{\sqrt{{\left|\Delta \right|}^{2}-E^{2}}} & |E|<\Delta \\ \frac{\pm \Delta }{\sqrt{E^{2}-{\left|\Delta \right|}^{2}}} & |E|>\Delta \; \end{array}\right.,$$

whereas the kinetic component, assuming that the SC is a macroscopic system at finite temperature *T*, is given by Equation (17) (see Equation (A29) and Appendix A for details).

(17) $${\hat{G}}_{SC}^{K}\left(E\right)=\left[{\hat{G}}_{SC}^{R}\left(E\right)-{\hat{G}}_{SC}^{A}\left(E\right)\right]\tanh\left(\frac{E}{2k_{B}T}\right).$$

### Non-Equilibrium Green’s Function in the Floquet Basis

At non-zero bias, the SC Green’s function depends on time due to the Josephson phase ϕ(t) of the order parameter Equation (6), via the transformation

(18) $${\hat{\tilde{G}}}_{SC}^{\lambda }\left(t,t^{\prime }\right)=\exp\left[i\frac{\phi \left(t\right){\hat{\tau }}_{0}⊗{\hat{\tau }}_{3}}{2}\right]{\hat{G}}_{SC}^{\lambda }\exp\left[-i\frac{\phi \left(t^{\prime }\right){\hat{\tau }}_{0}⊗{\hat{\tau }}_{3}}{2}\right],$$

with λ=R,K,A. Moreover, the time-dependence of the Josephson phase of the normal SC lead in Equation (6), determines a periodic time-dependence on the Green’s functions, and hence a Floquet representation [33] in Fourier space is the natural choice. Moreover, when Fourier transforming to the energy domain it is clear that, as a consequence of the voltage dependence of the Josephson phase, the Green’s functions acquire an energy shift 2eV, which we express as

(19) $${\hat{G}}_{SC}^{\lambda }\left(E+2eVm,E+2eVn\right)\equiv {\left[{\hat{G}}_{SC}^{\lambda }\left(E\right)\right]}_{mn}.$$

Each ${\hat{G}}_{SC}^{\lambda }$, for λ=A,R,K, is a 4×4 matrix, with indices m,n=1,…,4(2D+1). This motivates the introduction of new indices *M*, *N* to designate the matrix elements associated to the Floquet basis in each of the Green’s function components R,K,A

(20) $$M=\mathrm{integer}\left[\frac{m-1}{4}\right]\;,\;N=\mathrm{integer}\left[\frac{n-1}{4}\right],$$

with $E_{N}=E-2eV\left(D-N\right)$, and *D* representing the Floquet mode. Therefore, the superconductor Green’s function in the Floquet basis is a block matrix expressed as

(21) $${\left[{\hat{G}}_{SC}^{\lambda }\left(E\right)\right]}_{mn}={\left[{\hat{G}}_{SC}^{\mathrm{diag}}\left(E\right)\right]}_{mn}+{\left[{\hat{G}}_{SC}^{+}\left(E\right)\right]}_{mn}+{\left[{\hat{G}}_{SC}^{-}\left(E\right)\right]}_{mn},$$

where

$${\left[{\hat{G}}_{SC}^{\mathrm{diag}}\left(E\right)\right]}_{mn}=\delta_{M,N}{\left(\begin{array}{cccc} A(E_{N}-eV) & & & \\ & -A(E_{N}+eV) & & \\ & & A(E_{N}+eV) & \\ & & & -A(E_{N}-eV) \end{array}\right)}_{m-4N,n-4N}$$

$${\left[{\hat{G}}_{SC}^{-}\left(E\right)\right]}_{mn}=\delta_{M,N+1}{\left(\begin{array}{cccc} {}[cc|cc]0 & B(E_{N}+eV) & & \\ 0 & 0 & & \\ & & 0 & 0\\ & & -B(E_{N}+eV) & 0 \end{array}\right)}_{m-4(N+1),n-4N}$$

$${\left[{\hat{G}}_{SC}^{+}\left(E\right)\right]}_{mn}=\delta_{M,N-1}{\left(\begin{array}{cccc} {}[cc|cc]0 & 0 & & \\ B(E_{N}-eV) & 0 & & \\ & & 0 & -B(E_{N}-eV)\\ & & 0 & 0 \end{array}\right)}_{m-4(N-1),n-4N}.$$

Taking functional derivatives of the generating functional with respect to the source terms (see Appendix A), we obtain the current flowing across the nano-junction

(22) $$\begin{array}{ccc} J\left(T,V\right)\equiv \langle \hat{J}\left(T,V\right)\rangle & = & {\left.\frac{\delta Z_{C}\left[\alpha \right]}{\delta \alpha (t)}\right|}_{\alpha \to 0}\\ & = & \frac{e|t_{R}{|}^{2}{\left|t_{L}\right|}^{2}}{8}\mathrm{Tr}\int \;dE{\hat{G}}_{TS}\left(E\right){\hat{\Lambda }}^{T}\left\{{\hat{G}}_{QD}\left(E\right){\hat{M}}_{030}{\hat{G}}_{SC}\left(E\right){\hat{M}}_{100}{\hat{G}}_{QD}\left(E\right)\right.\\ & \; & -\left.{\hat{G}}_{QD}\left(E\right){\hat{M}}_{100}{\hat{G}}_{SC}\left(E\right){\hat{M}}_{030}{\hat{G}}_{QD}\left(E\right)\right\}\hat{\Lambda }, \end{array}$$

where $\hat{\Lambda }=\left({\hat{\sigma }}_{0}⊗\left(\left({\hat{\tau }}_{3}⊗{\hat{s}}_{0}\right)\cdot V\right)\right)$ and Tr implies taking the trace over Keldysh and Floquet subspaces, respectively. Similarly, the energy flux through the nano-junction is given by the expression

(23) $$\begin{array}{ccc} U(T,V) & = & \frac{|t_{R}{|}^{2}{\left|t_{L}\right|}^{2}}{8}\mathrm{Tr}\int \;dE\;E\;{\hat{G}}_{TS}\left(E\right){\hat{\Lambda }}^{T}\left\{{\hat{G}}_{QD}\left(E\right){\hat{M}}_{030}{\hat{G}}_{SC}\left(E\right){\hat{M}}_{100}{\hat{G}}_{QD}\left(E\right)\right.-\\ & \; & \left.{\hat{G}}_{QD}\left(E\right){\hat{M}}_{100}{\hat{G}}_{SC}\left(E\right){\hat{M}}_{030}{\hat{G}}_{QD}\left(E\right)\right\}\hat{\Lambda }. \end{array}$$

## 4. Results

### 4.1. Electrical Current

**Figure 2:**
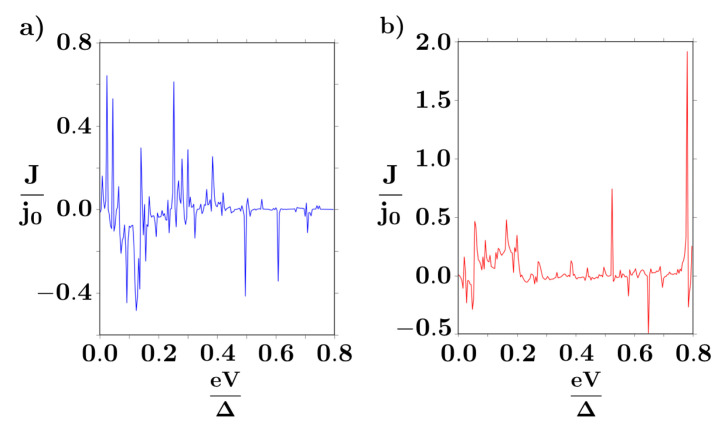
Tunneling current through the nano-junction for zero (**a**) and H=0.3Δ (**b**) magnetic field. We set $\epsilon =-0.01\Delta ,T=0.1\Delta /k_{B}$ and the coupling constants $\Gamma_{L}=0.7\Delta$ and $\Gamma_{R}=0.05\Delta$ with $j_{0}=e\Delta /2\hbar$. Here Δ represents the BCS order parameter, chosen as the natural energy scale in the model.

**Figure 3:**
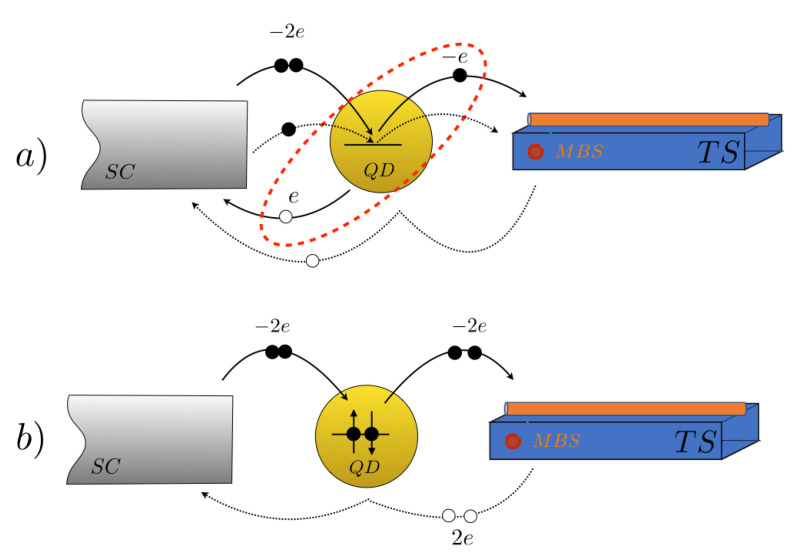
(**a**) A Cooper pair suffers an AR at the interface with the QD (red dashed line), and two AR’s at the TS interface and at the SC interface (dotted line) respectively, thus generating Andreev bound states; (**b**) The Cooper pair splits into two electrons of opposite spin, in order to occupy the dot’s level, thus producing two backscattered holes at the TS interface. The AR of an electron (hole) is equivalent to the transfer of a single Cooper pair in (out) of the superconducting condensate.

For the current *J* - voltage *V* characteristics, we considered a bias in the range eV<Δ, with Δ the BCS gap of the normal superconductor. The total number *n* of Floquet modes to be included in the numerical evaluation of the formulas was adjusted such that the calculated electrical current converged. In our case, we found that the first n=6 Floquet modes were sufficient to achieve the convergence criteria. The contribution of the first n=0 mode is notoriously small because the n=0 Green’s function has only diagonal elements, with the off-diagonal elements being present only for the n≥1 Floquet modes. The current through the nano-junction is shown in Figure 2, where the oscillating behavior as a function of bias *V* is a consequence of the presence of multiple Andreev reflections (see Figure 3), responsible for tunneling from the quantum dot to the non-trivial topological superconductor.

The current is notoriously sensitive to small changes in the externally applied magnetic field. Indeed, the Zeeman energy splitting $H=g\mu_{B}h$ of the dot energy level contributes to a local spin filtering effect for the current, as seen in the highest peak in the inset of Figure 2. For small magnetic fields, the principal peak will be present, varying smoothly its position, but for H>0.3Δ the highest Zeeman-splitted level will not be reachable for electron states at the dot, thus reducing the transport rate. As a consequence, configurations with single electronic occupation at the dot dominate transport in this high-field regime in favour of double occupation, as verified from the local dot’s occupation number

**Figure 4:**
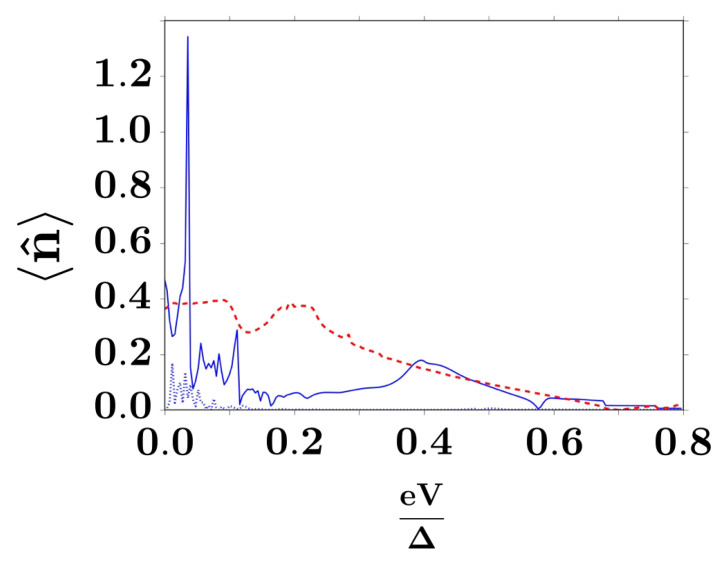
Occupation number for different values of the magnetic field *h*, with effective Zeeman coupling $H=g\mu_{B}h$. The curves represent the dot occupation at H=0 (dotted line), H=0.1Δ (solid line) and H=0.3Δ (dashed line), respectively, with Δ the BCS order parameter, chosen as the natural energy scale in the model.

(24) 〈n^QD〉=Tr∫dEnF(E,V,T)−1πIm{G^QDR(E,V,T},

where $n_{F}\left(E,V,T\right)={\left(\exp[\left(E-eV\right)/k_{B}T]+1\right)}^{-1}$ is the Fermi distribution function. In Figure 4, we represent the bias dependence of the local occupation number at the quantum dot, at different values of the external magnetic field expressed in terms of the Zeeman splitting *H*.

### 4.2. Thermoelectric Performance

In order to study the thermoelectric properties of the nano-junction, we need to consider the temperature dependence of the electric current. We assume that the s-wave superconductor (L-lead) is in local thermal equilibrium at temperature $T_{L}=T$, while the topological superconductor (R-lead) is at zero temperature $T_{R}=0$, such that the thermal flux is generated by the temperature difference between the leads $\Delta T=T_{L}-T_{R}=T$. As before, we set $T<\Delta /k_{B}$ . Transport coefficients are usually defined within the linear response regime [23,27,29,30,34], where Onsager relations [23,26,27,30] and Green-Kubo [25,29,32] formulas can be applied. For non-equilibrium quantum systems with strong non-linearities, however, it is possible to define generalizations of those transport coefficients [24]. In particular, for electric transport in non-linear systems it is customary to define the differential conductance [24,35,36]

(25) $$\begin{array}{c} \sigma \left(T,V\right)={\left.\frac{\partial J}{\partial V}\right|}_{T}. \end{array}$$

For thermal transport coefficients, starting from the entropy flux $J_{\Sigma }\left(T,V\right)=T^{-1}U\left(T,V\right)-T^{-1}\mu e^{-1}J\left(T,V\right)$, it is possible to define a thermal flux $J_{Q}=TJ_{\Sigma }$, such that differential thermal conductance in the nonlinear response regime [24] becomes (see Appendix B for details)

(26) $$\begin{array}{c} \kappa \left(T,V\right)={\left.\frac{\partial J_{Q}}{\partial T}\right|}_{J=0}={\left.\frac{\partial U}{\partial T}\right|}_{J=0}. \end{array}$$

The condition of vanishing electrical current J(T,V)=0 involved in the definition of the thermal conductance, establishes an implicit (nonlinear) relation between the temperature difference and the bias voltage across the junction, i.e., ${\left.V(T)\right|}_{J=0}$. Therefore, applying the implicit function theorem for partial differentiation, we have that under the condition J(T,V)=0

(27) $$\begin{array}{c} {\left.\frac{\partial }{\partial T}J\left(T,V\right)\right|}_{J=0}=0={\left.\frac{\partial J}{\partial T}\right|}_{V}+{\left.\frac{\partial J}{\partial V}\right|}_{T}{\left.\frac{\partial V}{\partial T}\right|}_{J=0} \end{array}$$

Solving from the equation above, we obtain a natural generalization of the Seebeck coefficient to the nonlinear response regime [24],

(28) $$S\left(T,V\right)={\left.-\frac{\partial V}{\partial T}\right|}_{J=0}=\frac{{\left.\frac{\partial J}{\partial T}\right|}_{V}}{{\left.\frac{\partial J}{\partial V}\right|}_{T}}.$$

The differential conductance in Equation (25) follows an operational definition that trivially matches the linear response value in the limit V→0. For the generalized nonlinear Seebeck coefficient and thermal conductance, despite they clearly match the standard definitions in the linear response limit V→0 [24,27,28,37], a more careful analysis is required to justify them from the perspective of entropy flow and production in the context of irreversible thermodynamics [23,24,26,30], as presented in Appendix B.

The thermoelectric performance of the nano-junction is represented by the thermoelectric figure of merit, commonly expressed by the dimensionless quantity [20,21,27,37,38]

(29) $$ZT=\frac{S^{2}\sigma }{\kappa },$$

where σ(T,V) and κ(T,V) are the electrical and thermal differential conductances defined by Equations (25) and (26), respectively, and S(T,V) is the Seebeck coefficient calculated from Equation (28).

**Figure 5:**
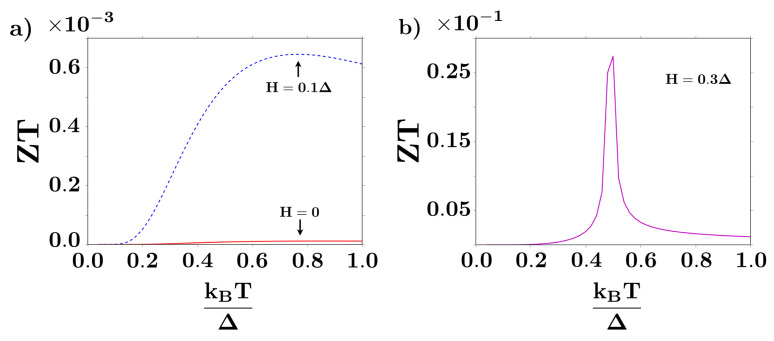
Thermoelectric figure of merit as a function of temperature at a fixed bias of 0.7 eV/Δ, at different magnetic fields *h*, with $H=g\mu_{B}h$ the effective Zeeman coupling (**a**). Notice the sharp response at $T∼0.5\Delta /k_{B}$ at an applied magnetic field H=0.3Δ (**b**). Here Δ represents the BCS order parameter, chosen as the natural energy scale in the model.

Figure 5 shows the thermoelectric figure of merit of the junction for different bias voltages, reaching its maximum at 0.7 eV/Δ when H=0.3Δ (the inset). The magnetic field breaks the electronic spin symmetry, giving rise to different energy contributions for spin up or spin down fermions: electrons with a spin parallel to the field dominate in the whole transport process as compared to those with anti-parallel spin, thus leading to a spin-filtering effect in the current through the junction. This filtering effect is reflected on the sharp thermal response of the figure of merit at finite magnetic fields, as seen in Figure 5. Until now it has been very hard to achieve high ZT in thermoelectric devices, because σ,κ and *S* cannot be independently controlled in the linear response regime: a material with large electrical conductivity σ has a large thermal conductivity κ when the Wiedemann-Franz law applies [24,27,37]. It is possible, however, to achieve some decoupling in materials where electron-phonon scattering is weak, while phonon-phonon scattering is high [19,20,21,22]. In this system, the comparatively small values of ZT are a consequence of the Seebeck coefficient of the device, due to the weak dependence of the electrical current on the temperature (see Equation (28)). This feature is not so surprising, considering that the model does not involve lattice phonons besides the BCS Hamiltonian for the normal s-wave superconductor, where they are implicitly involved through the BCS gap Δ.

We remark the strong and sharp response of the thermoelectric performance of the system as a function of temperature, when H>0, as seen for instance in Figure 5b. We propose that this interesting feature can be applied for thermal sensing with nano-metric spatial resolution in microelectronics and future nano-electronics, in order to mitigate and prevent the deleterious effects of excessive Joule heating in device components.

## 5. Discussion

Along this work, we studied the non-equilibrium electric and thermal transport through a topological nano-junction, in the context of the Keldysh formalism, by exploring the non-linear response of the junction at different applied magnetic fields. Due to the diversity of quasi-particle excitations present in the system, such as Majorana fermions and Cooper pairs, the electrical current presents a high nonlinear behavior as a characteristic signature of virtual processes known as multiple Andreev reflections between the leads and the quantum dot (see Figure 3), in agreement with the literature [7,8]. The transport coefficients in the nonlinear regime are defined as generalizations of the ones in linear response, closely following the analysis in [24]. In particular, the definition of thermal transport coefficients are tied to the notion of entropy production and fluxes through the junction. We have provided a detailed discussion on the implication of those definitions in the context of classical Onsager reciprocal relations [23,26,30], particularly on the restrictions imposed by the positive-definiteness of the entropy production rate [23,24,26,27,30] (see Appendix B for details).

**Figure 6:**
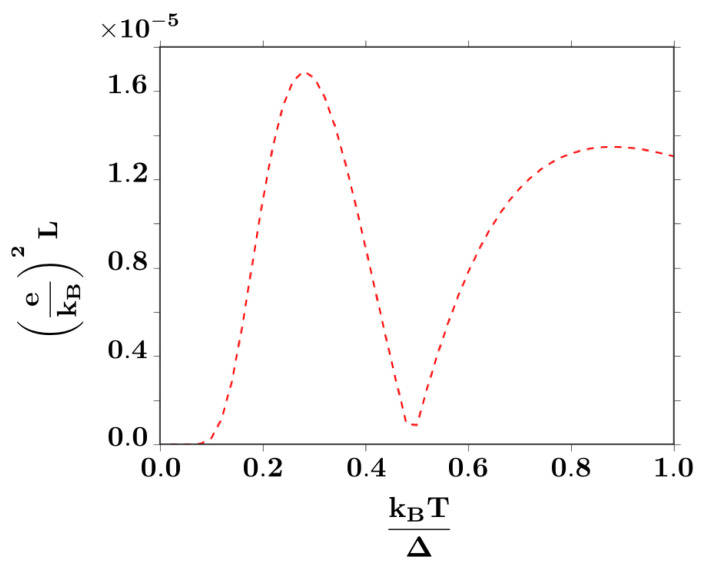
Lorenz number for H=0.3Δ. The minimum at $T∼0.5\Delta /k_{B}$ coincides with the sharp response in the thermoelectric figure of merit in Figure 5.

As seen in Figure 6, the Lorenz number for this system strongly differs from the Wiedemann-Franz law, which is only valid at low temperatures and in the linear-response regime [27,37]. On the first hand, as stated in Section 3, the junction corresponds to a system which is far from the linear response regime, due to the oscillatory effect of the bias voltage on the Josephson phase of the BCS superconducting lead. Therefore, the system is far from the domain of applicability of Wiedeman-Franz law [24,27,37]. In addition, as depicted in Figure 3, the multiple Andreev reflections tend to decouple the net particle flow from the electric charge flow. While the energy flux U(T,V) is always in the same direction as the particle flux, the charge flow may have the opposite direction. Consider for instance the process depicted in Figure 3b, where an electron pair flows from left to right, representing two quanta of negative charge in this direction, while a pair of positively charged holes is backscattered in the opposite direction: The net charge flow associated to this process is equivalent to four quanta of negative charge transported from left to right. However, the energy carried by electrons and holes, that determines the heat flux associated to the process, moves in counterflow for both types of particles. This decoupling between the electric and thermal flux directions is what leads to an extremely nontrivial behavior of the Lorenz number, thus exhibiting strong deviations from the Wiedemann-Franz law.

The applied magnetic field imposes a filtering effect over the electronic spin components of the current, thus suppressing transport of the antiparallel spin component at sufficiently strong fields. The magnetic field also contributes to the thermoelectric efficiency: at higher magnetic fields, the higher the electrical-heat conversion efficiency. We reported a maximum efficiency of $0.28\Delta /k_{B}$ at a bias of 0.7 eV/Δ, for an applied magnetic field of H=0.3Δ. We remark that the sharp thermoelectric response obtained at finite magnetic fields, could be used to construct a highly sensitive temperature sensor based on this topological nano-junction. A nanoscale thermocouple with nanometer-scale resolution may be an important contribution to mitigate and prevent the deleterious effects of excessive Joule heating in nano and micro-device components. 

## Figures and Tables

**Figure A1:**
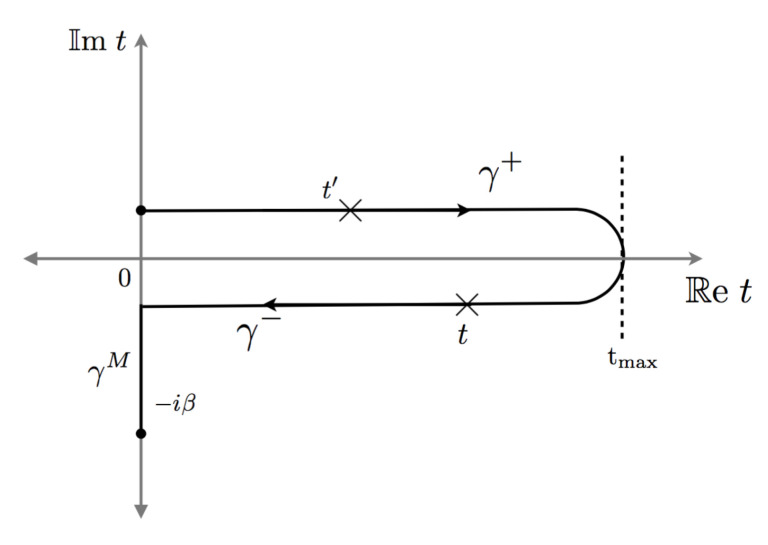
Contour $C=\gamma^{+}⊕\gamma^{-}⊕\gamma^{M}$, considering $t_{0}=0$.

**Figure A2:**
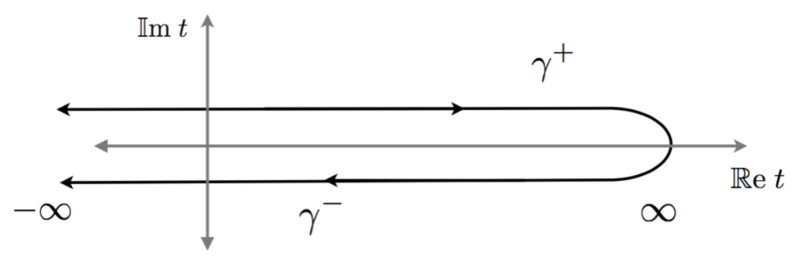
Keldysh contour $C=\gamma^{+}⊕\gamma^{-}$, considering $t_{0}\to -\infty$ and $t_{max}\to +\infty$.

## Appendix A. Non-Equilibrium Green’s Function Method

Along this appendix, we provide a brief but comprehensive review of the non-equilibrium Green’s functions formalism, particularly the Keldysh functional integral approach [31,32,39], which has demonstrated to be an effective method to deal with transport processes and other strongly correlated quantum phenomena [24,28,36].

### Appendix A.1. Closed-Time Contour Formalism

Let us first consider a general quantum system governed by the time-independent Hamiltonian

(A1) $$\hat{H}={\hat{H}}_{0}+{\hat{H}}_{I},$$

where ${\hat{H}}_{0}$ corresponds to the free particle Hamiltonian and ${\hat{H}}_{I}$ represents all the many-body interactions and static potentials acting upon the system. For $t<t_{0}$, we assume that the system is in thermal equilibrium and therefore it is characterized by an equilibrium density matrix operator

(A2) $${\hat{\rho }}_{0}=\sum_{\nu }p_{\nu }|\psi_{\nu }\rangle \langle \psi_{\nu }|=\frac{e^{\beta \hat{H}}}{Z_{H}}=\frac{e^{-\beta \hat{H}}}{\mathrm{Tr}(e^{-\beta \hat{H}})},$$

where $\beta =\frac{1}{k_{B}T}$ is the inverse temperature and $Z_{H}$ the canonical partition function. We further assume that at time $t=t_{0}$, the system is disconnected from the thermal reservoir and exposed to a time-dependent perturbation, such that the unitary time evolution of the system from its initial state is governed by the time-dependent Hamiltonian

(A3) $$H\left(t\right)=\hat{H}+{\hat{H}}^{\prime }\left(t\right),$$

and represented by the density matrix operator $\hat{\rho }\left(t\right)$, with the initial condition $\hat{\rho }\left(t=t_{0}\right)={\hat{\rho }}_{0}$. The density matrix operator follows a unitary evolution in time,

(A4) $$\hat{\rho }\left(t\right)={\hat{U}}_{H}\left(t,t_{0}\right)\hat{\rho }\left(t_{0}\right){\hat{U}}_{H}^{†}\left(t,t_{0}\right),$$

where the evolution operator corresponding to the time-dependent Hamiltonian H(t) is defined as

(A5) $${\hat{U}}_{H}\left(t,t_{0}\right)=\left\{\begin{array}{ccc} \hat{T}\exp\left[-\frac{i}{\hbar }\int_{t^{\prime }}^{t}\;d\bar{t}\hat{H}\left(\bar{t}\right)\right] & \mathrm{for} & t>t^{\prime }\\ \hat{\tilde{T}}\exp\left[-\frac{i}{\hbar }\int_{t^{\prime }}^{t}\;d\bar{t}\hat{H}\left(\bar{t}\right)\right] & \mathrm{for} & t<t^{\prime } \end{array}\right.,$$

and $\left(\hat{\tilde{T}}\right)\hat{T}$ denotes the (anti-) time ordering operator.

The time-dependent expectation value of an operator $\hat{O}$ representing a physical observable for $t>t_{0}$ is given by

(A6) $$\langle \hat{O}\left(t\right)\rangle =\mathrm{Tr}\left(\hat{\rho }\left(t\right)\hat{O}\right)=\frac{1}{Z_{H}}\mathrm{Tr}\left[e^{-\beta \hat{H}}{\hat{O}}_{H}\left(t,t_{0}\right)\right].$$

Considering that the Boltzmann exponential operator in the equilibrium density matrix can be written as

(A7) $$e^{-\beta \hat{H}}=e^{-\frac{i}{\hbar }\int_{t_{0}}^{t_{0}-i\beta }\;d\bar{t}\hat{H}}={\hat{U}}_{H}\left(t_{0}-i\beta ,t_{0}\right),$$

the expectation value in Equation (A6) has the form

(A8) $$\langle \hat{O}\left(t\right)\rangle =\frac{\mathrm{Tr}\left({\hat{U}}_{H}\left(t_{0}-i\beta ,t_{0}\right){\hat{U}}_{H}\left(t_{0},t\right)\hat{O}{\hat{U}}_{H}\left(t,t_{0}\right)\right)}{Z_{H}}.$$

By noticing the sequence of operators in Equation (A8) from right to left, it is straightforward to see that they follow the time ordering of $t_{0}\to t\to t_{0}\to t_{0}-i\beta$. This motivates the introduction of the Konstantinov-Perel [39] contour C with three branches: $\gamma^{+}:t_{0}\to t_{\max}$, $\gamma^{-}:t_{\max}\to t_{0}$ and $\gamma^{M}:t_{0}\to t_{0}-i\beta$, as is shown in Figure A1.

Noticing that the time-dependent contribution ${\hat{H}}^{\prime }\left(t\right)$ along the forward $\left(\gamma^{+}\right)$ and backward $\left(\gamma^{-}\right)$ contour cancels if no other operator is inserted, we can redefine the contour of ${\hat{U}}_{H}\left(t_{0}-i\beta ,t_{0}\right)$ in Equation (A8) to the corresponding action of the full time-dependent Hamiltonian $\hat{H}\left(t\right)$:

(A9) $${\hat{U}}_{H}\left(t_{0}-i\beta ,t_{0}\right)\to {\hat{U}}_{H}\left(t_{0}-i\beta ,t_{0}\right).$$

The fundamental object in any many-body theory is the single-particle Green’s function, which contains the information about single-particle excitations and their statistical distribution. The non- equilibrium Green’s function is defined as the contour-ordered expectation value

(A10) $$G\left(t,t^{\prime }\right)=-i\langle {\hat{T}}_{C}{\hat{c}}_{\alpha }\left(t\right){\hat{c}}_{\beta }^{†}\left(t^{\prime }\right)\rangle ,$$

where $\left({\hat{c}}^{†}\right)$$\hat{c}$ corresponds to the (creation-) annihilation operator, $t,t^{\prime }\in C$ and α,β may represent any internal degree of freedom. Due to the three branches of the contour C, the non-equilibrium Green’s function can be expressed conveniently as a 3×3 matrix [39]

(A11) $$\hat{G}=\left(\begin{array}{ccc} G_{++} & G_{+-} & G_{+M}\\ G_{-+} & G_{--} & G_{-M}\\ G_{M+} & G_{M-} & G_{MM} \end{array}\right),$$

where each component $G_{ij}$, with ij={+,−,M}, represents the position of times $t,t^{\prime }$ on each of the three different branches of C.

### Appendix A.2. Non-Equilibrium Keldysh Formalism

A simpler approach to describe non-equilibrium scenarios is the Keldysh formalism [31,32,39], where it is assumed that an interaction is adiabatically turned on from $t_{0}\to -\infty$, and the time evolution is extended to $t_{\max}\to \infty$. As a consequence, the imaginary-time contour $\gamma^{M}$ is decoupled from the contour C, thus neglecting correlations between initial states and evolving states [32,39] and restricting the time evolution to the real contour $\gamma^{+}⊕\gamma^{-}$. In steady state situations, initial correlations are expected to disappear for evolution times longer than the relaxation times of the system, making the Keldysh approximation a suitable formalism to deal with these problems [31,32,39]. Using these assumptions, the expectation value in Equation (A8) acquires the form

(A12) $$\langle \hat{O}\left(t\right)\rangle =\frac{1}{Z_{H}}\mathrm{Tr}\left[{\hat{U}}_{H}\left(t_{0}-i\beta ,t_{0}\right){\hat{U}}_{H}\left(t_{0},t\right)\hat{O}{\hat{U}}_{H}\left(t,t_{0}\right)\right].$$

Using the semi-group property of the unitary time evolution operator

(A13) $${\hat{U}}_{H}\left(t_{0},t\right)={\hat{U}}_{H}\left(t_{0},t_{\max}\right){\hat{U}}_{H}\left(t_{\max},t\right),$$

and taking the limits $\left(t_{0},t_{\max}\right)\to \left(-\infty ,\infty \right)$ one obtains

(A14) $$\langle \hat{O}\left(t\right)\rangle =\frac{1}{Z_{H}}\mathrm{Tr}\left[{\hat{U}}_{H}\left(-\infty ,\infty \right){\hat{U}}_{H}\left(\infty ,t\right)\hat{O}{\hat{U}}_{H}\left(t,-\infty \right){\hat{\rho }}_{0}\right].$$

The contour-ordered Keldysh Green’s function is now a subspace of Equation (A11), represented by the 2×2 matrix [25,32,39]

(A15) $$\hat{G}=\left(\begin{array}{cc} G_{++} & G_{+-}\\ G_{-+} & G_{--} \end{array}\right),$$

acting on the Keldysh contour as described in Figure A2.

In order to obtain expectation values from Equation (A12), it is convenient to modify the time-dependent Hamiltonian in a non-trivial way by adding a source term α(t)

(A16) $$\hat{H}\left(t\right)\to \hat{H}\left(t\right)\pm \hat{O}\alpha \left(t\right).$$

Here, the plus (minus) sign refers to the forward (backward) contour, respectively, making the time-evolution along the two branches no longer symmetric [32].

In this work, we will adopt the modern framework of many-body physics out of equilibrium, which corresponds to the integral formulation of quantum field theory. Consequently, we construct a generating functional given by [24,32,36]

(A17) $$Z_{C}\left[\alpha \right]\equiv \frac{\mathrm{Tr}\left({\hat{U}}_{C}\left[\alpha \right]{\hat{\rho }}_{0}\right)}{Z_{H}}\overset{\alpha \to 0}{\to }1,$$

where ${\hat{U}}_{C}\left[\alpha \right]$ is the time evolution operator along the closed-time contour C, containing the information of the source term α(t).

Using this new generating functional, the expectation value of any observable is obtained by means of functional differentiation

(A18) $$\langle \hat{O}\left(t\right)\rangle ={\left.\frac{\delta }{\delta \alpha (t)}Z_{C}\left[\alpha \right]\right|}_{\alpha \to 0}.$$

In contrast to equilibrium field theory methods, where the expectation value is given by functional derivatives of the logarithm of the generating functional, on the Keldysh contour the presence of the logarithm is unnecessary thanks to Equation (A17).

In the continuum notation, the generating functional may be written in terms of continuum Grassmann variables or fermionic fields $\left(\bar{\psi }\left(t\right),\psi \left(t\right)\right)$, representing creation and annihilation operators, respectively, according to

(A19) $$Z_{C}\left[\alpha \right]=\int D\left[\bar{\psi }\left(t\right),\psi \left(t\right)\right]\exp\left(i\int_{C}\;dt\left[\bar{\psi }\left(t\right){\hat{G}}^{-1}\psi \left(t\right)\right]\right)=\int D\left[\bar{\psi }\left(t\right),\psi \left(t\right)\right]e^{iS[\bar{\psi }\left(t\right),\psi \left(t\right)]},$$

where $S[\bar{\psi }\left(t\right),\psi \left(t\right)]$ is the action containing all the information of the full time Hamiltonian H(t). In order to avoid the contour integration and work with real time variables, the contour integration is split into two time branches [32,39]

(A20) $$\int_{C=\gamma^{+}⊕\gamma^{-}}\;d\tau =\int_{-\infty }^{\infty }\;dt+\int_{\infty }^{-\infty }\;dt=\int_{-\infty }^{\infty }\;dt-\int_{-\infty }^{\infty }\;dt,$$

and hence the fields split into two components

(A21) $$\psi \left(t\right)\to \left(\begin{array}{c} \psi^{+}\left(t\right)\\ \psi^{-}\left(t\right) \end{array}\right),$$

where $\psi^{+}\left(t\right)$ and $\psi^{-}\left(t\right)$ reside on the forward and backward contour, respectively. As a consequence, the action is now written as

(A22) $$S\left[\bar{\psi }\left(t\right),\psi \left(t\right)\right]=\int_{-\infty }^{\infty }\;dt\bar{\psi }\left(t\right){\hat{\sigma }}_{3}{\hat{G}}^{-1}\psi \left(t\right),$$

where ${\hat{\sigma }}_{3}=\left(\begin{array}{cc} 1 & 0\\ 0 & -1 \end{array}\right)$ is the third Pauli matrix acting on the Keldysh space, containing the minus sign from the contour splitting operation in Equation (A20).

The components of the matrix Green’s function in Equation (A15) are not linearly independent, but by definition they satisfy the constraint

(A23) $$G_{++}\left(t,t^{\prime }\right)+G_{--}\left(t,t^{\prime }\right)=G_{+-}\left(t,t^{\prime }\right)+G_{-+}\left(t,t^{\prime }\right),$$

which allows us to eliminate one of the components. To this end, one introduces the so-called Keldysh rotation [32,40] for fermionic fields ψ, by defining new fields according to

(A24) $$\psi_{1}\left(t\right)=\frac{1}{\sqrt{2}}\left(\psi^{+}\left(t\right)+\psi^{-}\left(t\right)\right)\;;\;\psi_{2}\left(t\right)=\frac{1}{\sqrt{2}}\left(\psi^{+}\left(t\right)-\psi^{-}\left(t\right)\right),$$

while for the *bar* fields

(A25) $${\bar{\psi }}_{1}\left(t\right)=\frac{1}{\sqrt{2}}\left({\bar{\psi }}^{+}\left(t\right)-{\bar{\psi }}^{-}\left(t\right)\right)\;;\;{\bar{\psi }}_{2}\left(t\right)=\frac{1}{\sqrt{2}}\left({\bar{\psi }}^{+}\left(t\right)+{\bar{\psi }}^{-}\left(t\right)\right).$$

Performing these transformations, the fermionic Green’s function can be represented in its triagonal form [32,39]

(A26) $$\hat{G}\left(t,t^{\prime }\right)=\left(\begin{array}{cc} G^{R}\left(t,t^{\prime }\right) & G^{K}\left(t,t^{\prime }\right)\\ 0 & G^{A}\left(t,t^{\prime }\right) \end{array}\right),$$

where the three linearly independent Green’s functions $G^{R},G^{A},G^{K}$ are called retarded (*R*), advanced (*A*) and kinetic (*K*) Green’s functions, respectively. In the fermionic operator formalism, these Green’s functions are given by [31,32,39]

(A27) $$\begin{array}{ccc} G^{R}\left(t,t^{\prime }\right) & = & -i\theta \left(t-t^{\prime }\right)\langle {\left[\hat{c}\left(t\right),{\hat{c}}^{†}\left(t^{\prime }\right)\right]}_{+}\rangle ,\\ G^{A}\left(t,t^{\prime }\right) & = & i\theta \left(t^{\prime }-t\right)\langle {\left[\hat{c}\left(t\right),{\hat{c}}^{†}\left(t^{\prime }\right)\right]}_{+}\rangle ,\\ G^{K}\left(t,t^{\prime }\right) & = & -i\langle {\left[\hat{c}\left(t\right),{\hat{c}}^{†}\left(t^{\prime }\right)\right]}_{-}\rangle , \end{array}$$

where ${\left[,\right]}_{(+)-}$ denotes the (anti-)commutator. In addition, stationary states satisfy time translational invariance $G^{R/A/K}\left(t,t^{\prime }\right)=G^{R/A/K}\left(t-t^{\prime }\right)$, which allows for a Fourier representation of the Green’s functions. The imaginary part of the retarded and advanced Green’s functions gives the single-particle spectral function [32,39]

(A28) A(ω)=−1πImGR(ω)=1πImGA(ω),

which represents the distribution of quasi-particle excitations in frequency (Fourier) space. On the other hand, in thermal equilibrium situations, it can be shown that Equation (A28) leads to [32,39]

(A29) $$G^{K}\left(\omega \right)=\left[G^{R}\left(\omega \right)-G^{A}\left(\omega \right)\right]\tanh\left(\frac{\beta \omega }{2}\right),$$

which corresponds to the fluctuation-dissipation theorem for a quantum mechanical system [25,29,32,39].

## Appendix B. Thermoelectric Transport Coefficients and Onsager Relations

At finite temperature, electric and thermal transport are interconnected. At the classical level, this connection is expressed by Onsager reciprocal relations [23,26,27,37]. In this appendix, we shall discuss the generalization of the electric and thermal transport coefficients for a quantum mechanical system, in connection with Onsager’s relations, mainly following the formalism presented in Ref. [24].

Classical irreversible thermodynamics, according to Onsager’s perspective, assumes that fluxes and forces are linearly coupled [23,26,28,30]

(A30) $$J_{i}=\sum_{j}L_{ij}X_{j},$$

where $J_{i}$ and $X_{j}$ are the thermodynamic fluxes and forces, respectively, and $L_{ij}$ represent a matrix of phenomenological coefficients. In the presence of a temperature gradient ∇T and an electric field E=−∇V, the macroscopic charge J and energy U fluxes, respectively, are given by

(A31) $$\begin{array}{ccc} J & = & L_{EE}\left(-\nabla V\right)+L_{ET}\nabla T,\\ U & = & L_{TE}\left(-\nabla V\right)+L_{TT}\nabla T. \end{array}$$

In the absence of temperature gradients, the charge flux reduces to $J=L_{EE}\left(-\nabla V\right)=\sigma E$, where $\sigma =L_{EE}$ is the electrical conductivity. On the other hand, if the electric current through the system vanishes, then the electric field and thermal gradient are linearly coupled via

(A32) $$E=-\nabla V=-L_{EE}^{-1}L_{ET}\nabla T=S\nabla T.$$

Here, the proportionality factor is known as the Seebeck coefficient or thermopower

(A33) $$S=-L_{EE}^{-1}L_{ET}=-{\left.\frac{\partial V}{\partial T}\right|}_{J=0}.$$

For vanishing electric current, the thermal flux is given by the relation

(A34) $$\begin{array}{c} U=-(L_{TE}L_{EE}^{-1}L_{ET}-L_{TT})\nabla T=-\kappa \nabla T, \end{array}$$

from which it follows that the thermal conductivity is given by

(A35) $$\kappa =L_{TE}L_{EE}^{-1}L_{ET}-L_{TT}.$$

A similar hydrodynamic analysis can be applied in terms of the charge J(T,V) and energy U(T,V) fluxes across a nano-junction [24]. Taking the QD as an open system in contact with the macroscopic leads, the continuity equation in steady-state regime reads

(A36) $$\begin{array}{c} \partial_{t}\langle \hat{n}\rangle =e^{-1}J_{L}-e^{-1}J_{R}=0, \end{array}$$

where $J_{R/L}$ are the currents from the left (*L*) and right (*R*) leads, respectively. Therefore, in steady-state when there is no charge accumulation at the QD, we have $J_{R}=J_{L}=J\left(T,V\right)$ . Similarly, for the energy balance across the QD, we have that in steady-state

(A37) $$\begin{array}{c} \partial_{t}\langle E\rangle =U_{L}-U_{R}=0, \end{array}$$

thus leading to the condition $U_{L}=U_{R}=U\left(T,V\right)$ of no net accumulation of energy at the QD. Let us now analyze the process from the perspective of non-equilibrium thermodynamics. The continuity equation for entropy (Σ) flow across the QD reads [24]

(A38) $$\begin{array}{c} \partial_{t}\Sigma +J_{\Sigma }^{R}-J_{\Sigma }^{L}=P_{\Sigma } \end{array}$$

Here, the entropy fluxes from left ($J_{\Sigma }^{L}$) and right ($J_{\Sigma }^{R}$) leads are given by [24]

(A39) $$\begin{array}{ccc} T_{L}J_{\Sigma }^{L} & = & U_{L}-\mu_{L}e^{-1}J_{L}=U\left(T,V\right)-\mu_{L}e^{-1}J\left(T,V\right),\\ T_{R}J_{\Sigma }^{R} & = & U_{R}-\mu_{L}e^{-1}J_{R}=U\left(T,V\right)-\mu_{R}e^{-1}J\left(T,V\right), \end{array}$$

with $\mu_{L/R}$ the chemical potentials at the left and right leads, respectively, whose difference corresponds to the bias imposed across the junction, $\mu_{L}-\mu_{R}=eV$. The temperatures at each lead are $T_{L}=T$ and $T_{R}=T-\Delta T$, respectively, with $\Delta T=T_{L}-T_{R}$ the temperature difference across the nano-junction. The entropy production rate $P_{\Sigma }$ should, according to classical irreversible thermodynamics, be expressible as a positive-definite linear combination of generalized fluxes and forces, i.e., [23,24,26,27,28]

(A40) $$\begin{array}{c} P_{\Sigma }^{classical}=\sum_{i}X_{i}J_{i}. \end{array}$$

Notice that, under steady-state conditions, $\partial_{t}\Sigma =0$, direct substitution of Equations (A39) into (A38) leads to [24]

(A41) $$\begin{array}{c} P_{\Sigma }=-U\left(T,V\right)\Delta \left(\frac{1}{T}\right)+e^{-1}J\left(T,V\right)\Delta \left(\frac{\mu }{T}\right). \end{array}$$

Remarkably, this has precisely the same structure of the classical expression Equation (A40), but generalized to account for the nonlinear dependence of the energy and electric fluxes. In particular, notice that under the conditions where the thermal conductance and Seebeck coefficient are defined, that is when the electric current across the junction vanishes, J(T,V)=0, the entropy production rate reduces to the expression [24]

(A42) $$\begin{array}{c} {\left.P_{\Sigma }\right|}_{J=0}=-U\left(T,V\right)\Delta \left(\frac{1}{T}\right)=U\left(T,V\right)\frac{T_{L}-T_{R}}{T_{L}T_{R}}>0. \end{array}$$

Here, the inequality (positive entropy production) implies that the energy (heat) flow has the same direction as the temperature difference across the junction, in perfect agreement with the second law of thermodynamics in the context of irreversible processes [23,26,30]. Therefore, the definition of thermal transport coefficients such as the thermal conductance and Seebeck coefficient in this non-linear regime is possible without violating the laws of irreversible thermodynamics. In particular, we have that the heat fluxes from the left and right leads to the dot are defined from the entropy fluxes as [24]

(A43) $$\begin{array}{ccc} J_{Q}^{L} & = & T_{L}J_{\Sigma }^{L}=U\left(T,V\right)-\mu_{L}e^{-1}J\left(T,V\right),\\ J_{Q}^{R} & = & T_{R}J_{\Sigma }^{L}=U\left(T,V\right)-\mu_{L}e^{-1}J\left(T,V\right). \end{array}$$

The thermal conductance is thus defined, as in standard experimental situations, under conditions where the electric current vanishes J(T,V)=0, thus implying that $J_{Q}^{L}=J_{Q}^{R}=U\left(T,V\right)$, and hence [24]

(A44) $$\begin{array}{c} \kappa \left(T,V\right)={\left.\frac{\partial U}{\partial T}\right|}_{J=0}. \end{array}$$

We remark that this definition is fully consistent with the standard linear-response limit Equation (A35), and the non-linear heat flux satisfies the second law of thermodynamics as seen in Equation (A42). The condition of vanishing electric current J(T,V)=0 establishes an implicit functional relation between the bias voltage V(T) and temperature. We can extract such relation from the theorem of implicit function differentiation

(A45) $$\begin{array}{c} {\left.\frac{\partial }{\partial T}J\left(T,V\right)\right|}_{J=0}=0={\left.\frac{\partial J}{\partial T}\right|}_{V}+{\left.\frac{\partial J}{\partial V}\right|}_{T}{\left.\frac{\partial V}{\partial T}\right|}_{J=0}. \end{array}$$

Solving the equation above, we obtain a natural generalization of the Seebeck coefficient to the nonlinear response regime [24],

(A46) $$S\left(T,V\right)={\left.-\frac{\partial V}{\partial T}\right|}_{J=0}=\frac{{\left.\frac{\partial J}{\partial T}\right|}_{V}}{{\left.\frac{\partial J}{\partial V}\right|}_{T}}.$$

We remark that this definition is in full agreement with the standard one in the limit of linear response V→0, Equation (A33).
